# Single-cell transcriptomic insights into the immune heterogeneity of immune checkpoint inhibitors related organ toxicities

**DOI:** 10.3389/fimmu.2026.1823949

**Published:** 2026-06-09

**Authors:** Hao Wen, Yange Qi, Jianbo Song, Xia Yan

**Affiliations:** 1 Third Hospital of Shanxi Medical University, Shanxi Bethune Hospital, Shanxi Academy of Medical Sciences, Tongji Shanxi Hospital, Taiyuan, China; 2 Shanxi Provincial People’s Hospital Affiliated to Shanxi Medical University, Taiyuan, China; 3 Shanxi Bethune Hospital, Shanxi Academy of Medical Sciences, Third Hospital of Shanxi Medical University, Tongji Shanxi Hospital, Taiyuan, China

**Keywords:** cardiotoxicity, colitis, ICIS, immune heterogeneity, ScRNA-seq, translational medicine

## Abstract

Immune checkpoint inhibitors (ICIs) have revolutionized cancer therapy, but their increasing clinical use has led to an increasing incidence of immune-related adverse events (irAEs), among which colitis and cardiotoxicity are particularly severe, compromising both quality of life and therapeutic outcomes. Recent advances in single-cell RNA sequencing (scRNA-seq) have enabled high-resolution profiling of immune cell heterogeneity, offering new insights into the mechanisms of irAEs. By dissecting immune cell phenotypes and interactions across affected organs, scRNA-seq helps to clarify the pathogenesis of toxicity and supports the development of personalized immunomodulatory strategies. In this review, we summarize the clinical features, underlying mechanisms, and immune landscape of ICI-associated colitis and cardiotoxicity. Through comparative analysis, we highlight shared mechanisms-such as T cell clonal expansion, IFN-γ/IL-1β-driven inflammatory circuits, and JAK-STAT signaling-as well as organ-specific features, including microbiota-dependent regulation in colitis and autoantigen-initiated responses in myocarditis. We also explore emerging therapeutic targets and biomarkers identified by scRNA-seq and discuss their application in other irAEs, such as pneumonitis. Together, this work underscores the value of single-cell technologies in elucidating irAE heterogeneity, advancing irAEs management from reactive response to precise prevention, and guiding future translational research.

## 1 Introduction

The advent of immune checkpoint inhibitors (ICIs), particularly those targeting PD-1/PD-L1 and CTLA-4, has revolutionized cancer therapy (1). By releasing inhibitory brakes on T cells, ICIs restore antitumor immunity and have demonstrated remarkable clinical efficacy in melanoma, lung cancer, renal cell carcinoma, and other cancers (2). However, their success has been tempered by the emergence of immune-related adverse events (irAEs), which arise from unintended immune activation in healthy tissues (3). IrAEs may affect nearly any organ, with colitis and myocarditis representing two of the most clinically significant manifestations (4, 5). These toxicities not only impair quality of life but also necessitate treatment interruption or discontinuation, ultimately undermining long-term therapeutic benefit. The underlying mechanisms of irAEs remain incompletely understood, and their unpredictable onset underscores the need for enhanced mechanistic understanding.

A central concept in irAE development is immune heterogeneity-the variability in immune cell composition, phenotype, and functional state across individuals and tissues. This heterogeneity arises from a complex interplay of factors, including HLA(Human Leukocyte Antigen) genotype, gut microbiota, prior treatments, comorbidities, and tumor-intrinsic genomic features, and jointly determines both the response to ICIs and the risk of toxicity (6–9). Traditional transcriptomic approaches, such as bulk RNA sequencing, lack the resolution to interrogate this complexity (10). Single-cell RNA sequencing (scRNA-seq), as a new technique, can provide high-dimensional, cell-specific transcriptional profiles, enabling the identification of distinct immune subsets and dynamic changes over time (11–14). For example, in patients with non-small cell lung cancer, responders exhibit expansion of cytotoxic T and natural killer (NK) cells and enhanced antigen presentation, whereas nonresponders show increased immunosuppressive myeloid infiltration (15). These findings underscore the capacity of scRNA-seq to distinguish protective immune responses from pathogenic responses during ICI therapy (16). In addition to response prediction, scRNA-seq has also elucidated the mechanisms of resistance. In relapsed B-cell lymphoma, certain immune populations upregulate cytochrome P450 enzymes, potentially impairing drug efficacy (17). Moreover, even rare immune cell subsets, including tissue-resident cytotoxic T cells and proinflammatory myeloid populations, have been implicated in the pathogenesis of irAEs (18). These advances establish scRNA-seq as a critical tool for dissecting the immunopathology of ICI-induced toxicity.

**Figure 1:**
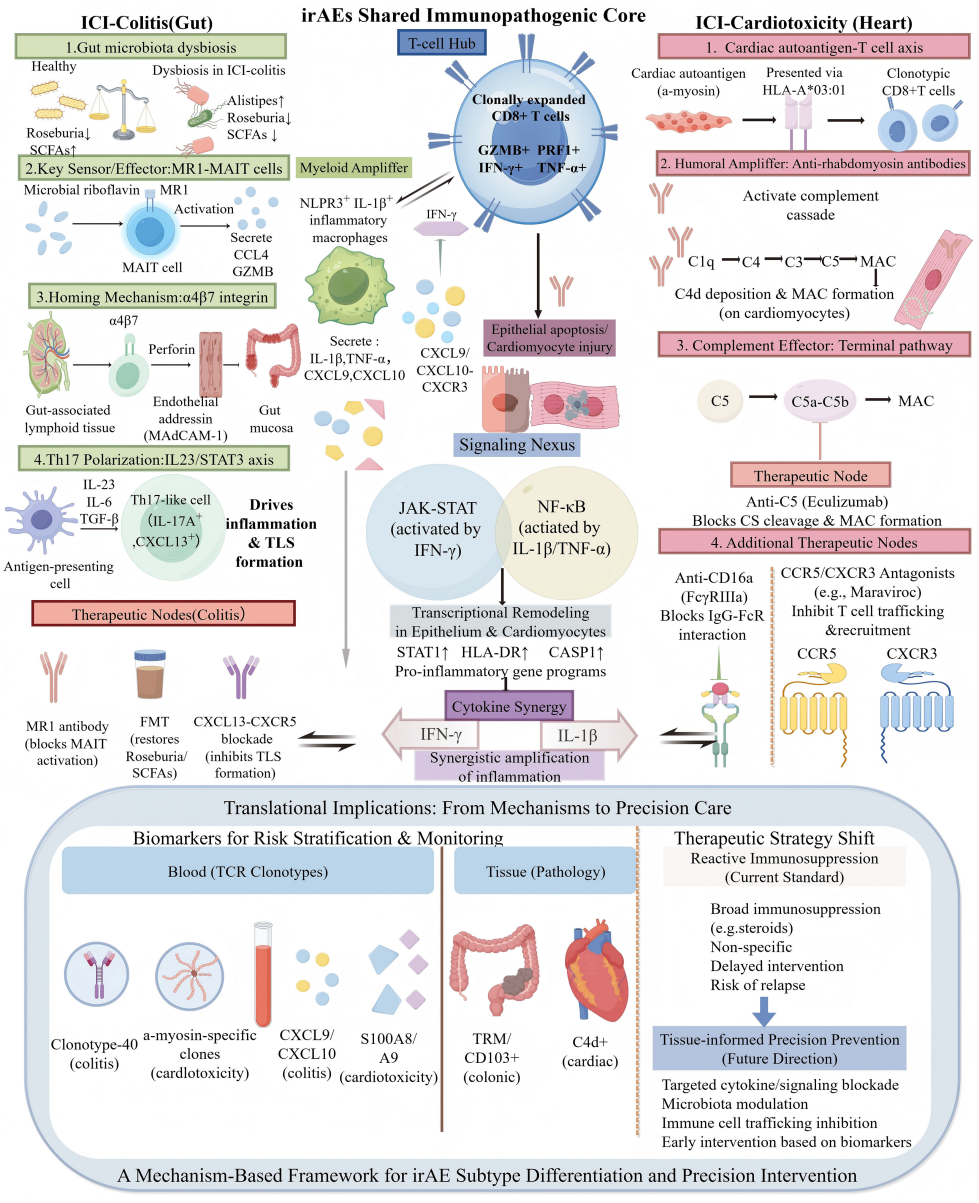
A mechanism-based framework for irAE subtype differentiation and precision intervention. This schematic illustrates the shared immunopathogenic core and distinct tissue-specific mechanisms underlying ICI-colitis (left, green) and ICI-MC (right, pink). The central T-cell hub highlights clonally expanded cytotoxic CD8^+^ T cells, GZMB^+^, PRF1^+^, IFN-γ^+^, TNF-α^+^ as the common driver, interacting with myeloid amplifiers and converging signaling nexus Tissue-specific pathways include: (1) for colitis—gut microbiota dysbiosis, MR1-MAIT cell activation, α4β7 integrin-mediated gut homing, and Th17 polarization via the IL-23/STAT3 axis driving tertiary TLS formation; (2) for cardiotoxicity—cardiac autoantigen, anti-rhabdomyosin antibody-mediated complement cascade activation (C1q→MAC), and terminal complement pathway. Therapeutic nodes for colitis include MR1 antibody blockade, fecal microbiota transplantation (FMT), and CXCL13-CXCR5 blockade; for cardiotoxicity, anti-C5 (Eculizumab), anti-CD16a, and CCR5/CXCR3 antagonists are proposed. The bottom panel presents translational implications, including blood-based biomarkers (TCR clonotypes, CXCL9/10, S100A8/A9) and tissue pathology markers TRM/CD103^+^, C4d^+^ for risk stratification, alongside a strategic shift from reactive broad immunosuppression to tissue-informed precision prevention.

In this review, we focus on colitis and myocarditis-two prototypical irAEs that illustrate both the common and divergent immune mechanisms underlying ICI toxicity. We summarize current understanding of their pathophysiology (**Figure 1**), highlight findings from scRNA-seq studies that have advanced our understanding of these conditions, and discuss how these insights inform the development of personalized therapeutic strategies and predictive biomarkers.

## 2 ICI-associated colitis

### 2.1 Clinical manifestations and diagnosis

ICI-associated colitis(ICI-colitis) is a frequent gastrointestinal irAE, the incidence of which varies depending on the ICI regimen. Notably, compared with anti-PD-1/PD-L1 therapies,anti-CTLA-4 monotherapy is associated with a greater risk of colitis, and combination regimens further increase this risk (19, 20). Advanced age has also been correlated with increased susceptibility (9). Multiple studies have reported that ICI-associated colitis occurs earlier with anti-CTLA-4 monotherapy or combined CTLA-4/PD-1 blockade than with anti-PD-1/PD-L1 monotherapy. The median onset is approximately 55–58 days for CTLA-4-containing regimens, compared with nearly 120 days for PD-1/PD-L1 blockade alone (21, 22), suggesting that CTLA-4 inhibition may be a major driver of early-onset colitis. Clinically, ICI-induced colitis typically presents as diarrhea (the predominant symptom), abdominal pain, hematochezia, and weight loss (23). Symptoms commonly arise between 4 and 8 weeks after initiating immunotherapy and may persist for weeks or even months. Diagnosis involves comprehensive clinical assessment, laboratory evaluations (such as inflammatory markers), and colonoscopic examination, which usually reveal mucosal inflammation characterized by erythema, edema, friability, and ulceration. Histopathological biopsy is essential for differentiating ICI-induced colitis from infections, graft-versus-host disease, or preexisting inflammatory bowel disease (24). Regardless of whether ICIs are administered as monotherapy or in combination, ICI-associated colitis is pathologically characterized by marked lymphocytic infiltration of the intestinal mucosa, particularly CD8^+^ cytotoxic T cells. These activated T cells can damage intestinal epithelial cells, resulting in epithelial barrier disruption, cryptitis, crypt abscesses, and, in severe cases, ulceration (25). Given the earlier onset of colitis in patients receiving combination therapy, gastrointestinal symptoms should be closely monitored from the initial weeks after treatment initiation. However, because onset varies widely, ranging from weeks to months and occasionally occurring after treatment discontinuation, long-term follow-up remains necessary for all patients treated with ICIs.

### 2.2 Pathophysiologic mechanisms

The pathogenesis of ICI-induced colitis remains incompletely understood, but accumulating evidence highlights crucial roles for immune dysregulation and alterations in the composition of the gut microbiota.

The intestine is continuously exposed to a broad range of exogenous antigens, including dietary components and commensal microorganisms, and therefore relies on a tightly regulated immune balance. ICIs disrupt this intrinsic intestinal tolerance by releasing inhibitory constraints on T cells, thereby promoting aberrant immune responses against intestinal self-antigens or microbiota-derived antigens (26–28). Anti-PD-1/PD-L1 agents relieve immune suppression within peripheral tissues by blocking ligand interactions involving PD-L1 expressed on intestinal epithelial cells and antigen-presenting cells. ScRNA-seq analysis revealed that the pathological core of colitis patients receiving anti-PD-1 monotherapy lies in the activation of intrinsic immune cells within the colonic mucosa, particularly the deregulation of Tissue-resident memory T cells (TRM). CD8^+^ TRM cells are a type of T cell that permanently resides in non-lymphatic tissues (such as the intestinal mucosa), playing a crucial role in local immune surveillance and rapid response to secondary infections. Under steady-state conditions, their activity is strictly controlled by various inhibitory signals, including PD-1. Following anti-PD-1 treatment, CD8^+^ TRM cells are rapidly activated, and their transcriptomic characteristics undergo significant changes, transitioning from a resting state to highly proliferative and cytotoxic effector cells. These activated cells upregulate genes related to proliferation and cytotoxic effector molecules (such as GZMB, PRF1), becoming the primary factors attacking the intestinal epithelium. Additionally, an increased polarization of CD4^+^ T cells towards the Th1 phenotype was observed, which may further amplify and support the cytotoxic response of CD8^+^ T cells by secreting cytokines such as IFN-γ. On this basis, combination therapy further blocks the CTLA-4 pathway. CTLA-4 primarily regulates the early phase of T-cell activation by competing with CD28 for binding to B7 molecules, including CD80 and CD86, on antigen-presenting cells. Because CTLA-4 has a higher binding affinity than CD28, it raises the threshold for T-cell activation. Accordingly, anti-CTLA-4 therapy may broaden the repertoire and magnitude of activated T-cell clones, including low-affinity clones reactive to self-antigens. ScRNA-seq showed that combination therapy not only led to a sharp increase in the total number of CD8^+^ T cells, but more importantly, it specifically expanded several unique and potentially more pathogenic subsets of CD8^+^ T cells (CX3CR1^+^ CD8^+^T cells, GZMK^+^ CD8+T cells) (29–31). The gene expression profiles of these specific CD8^+^ T cell subsets are rich in genes related to TCR signaling, tissue homing, cytotoxicity, and pro-inflammatory response, indicating that they may be key effector cells in combination therapy for irColitis. In addition to enhancing T-cell activation, CTLA-4 blockade may also alter the abundance and suppressive function of regulatory T cells, further amplifying intestinal immune dysregulation (32, 33). Specifically, CTLA-4 inhibitors target CTLA-4 expressed on Tregs, facilitating their elimination through antibody-dependent cellular cytotoxicity, which is primarily mediated by NK cells (34). This depletion results in reduced peripheral tolerance, permitting unchecked activation of autoreactive effector T cells and subsequent mucosal inflammation. Similarly, PD-1/PD-L1 blockade reduces FoxP3 expression in Tregs, impairing their immunosuppressive function (35, 36). This impairment allows enhanced effector T-cell proliferation and activation, directly contributing to epithelial injury and inflammatory manifestations of colitis.

In parallel, the gut microbiota has emerged as a significant modulator of ICI-induced colitis. The composition and diversity of gut microbes profoundly influence mucosal immune responses and the balance between immune tolerance and inflammation. Certain microbial taxa, such as *Faecalibacterium prausnitzii* and Gemmiger within the Firmicutes phylum, have been correlated with increased susceptibility to colitis, whereas a greater abundance of Bacteroides species is associated with reduced risk (37, 38). In a study of metastatic melanoma patients treated with ipilimumab, individuals who did not develop colitis presented a microbiome enriched in Bacteroides and *F. prausnitzii*, suggesting protective roles for these species. Conversely, dysbiotic shifts induced by antibiotic exposure can markedly increase the risk of colitis by disrupting microbial equilibrium, highlighting the central role of the microbiota in intestinal homeostasissm (39).

Mechanistically, the microbiota modulates colitis through multiple interacting pathways. These include the production of metabolites such as short-chain fatty acids (SCFAs), polyamines, and vitamins, which reinforce epithelial barrier integrity and enhance mucosal immune tolerance (40). Beneficial commensals, notably *F. prausnitzii*, facilitate Treg differentiation and function, bolstering mucosal tolerance. In contrast, dysbiotic or pathogenic microbial species stimulate proinflammatory CD4^+^ T-cell responses, promoting IFN-γ secretion and enhancing effector T-cell recruitment and activation (41, 42). Furthermore, bacterial components such as lipopolysaccharides and exoenzymes directly activate innate immune pathways, exacerbating local inflammation. For example, Lactobacillus rhamnosus GG mitigates colitis severity through a Toll-like receptor 2-dependent dendritic cell pathway (43), exemplifying the therapeutic potential of targeted microbiome modulation.

Collectively, these findings highlight the intricate interplay between immune checkpoints, regulatory immune cell networks, and the intestinal microbiota in orchestrating the pathophysiological landscape of ICI-induced colitis.

### 2.3 Insights from single-cell RNA sequencing

#### 2.3.1 Immune heterogeneity and signaling cascades

By revealing the intricate heterogeneity and functional states of immune cell subsets in inflamed intestinal mucosa, scRNA-seq has substantially advanced our understanding of the immunopathogenesis of ICI-induced colitis.

Within the T-cell compartment, cytotoxic CD8^+^ effector T cells (Teffs) exhibit significant clonal expansion, accompanied by elevated expression of cytolytic mediators, including granzyme B (GZMB), granzyme K (GZMK), perforin (PRF1), and IFN-γ, which directly mediate epithelial damage (31, 44). A distinct CD8^+^ T-cell subset characterized by high expression of ITGB2 and trafficking markers (SELL, S1PR1) is especially enriched in patients treated with anti-PD-1/CTLA-4 combination therapy and is closely associated with disease severity. These cells likely originate from circulating precursors and migrate into the intestinal mucosa, where they exert pathogenic effects (29). TRMs, identified by high expression of CD103 (ITGAE), CD69, HLA-DR, and CD38, also exhibit marked expansion and sustained inflammatory activity, primarily through IFN-γ secretion and cytotoxic potential (e.g., GZMB and PRF1). T-cell receptor sequencing (TCR-seq) revealed approximately 56.6% clonotype overlap between TRMs and Teff cells (45), indicating a shared lineage origin or local differentiation from common precursor populations. These TRM and Teff populations act synergistically by secreting IFN-γ and TNF-α, which activate the JAK-STAT1 signaling pathway in epithelial cells. This leads to CASP1-mediated apoptosis, upregulation of HLA-DR, neutrophil infiltration, and crypt abscess formation (29, 45). In parallel, persistent IFN stimulation induces broader transcriptional remodeling within colonic epithelial cells. Specifically, epithelial cells upregulate immune-modulatory molecules such as PD-L1 and STAT1 while concurrently downregulating aquaporin-8 (AQP8), a key channel for fluid absorption—thereby contributing to diarrhea and mucosal dysfunction. Furthermore, IFN-driven expression of CX3CL1 enhances the recruitment of CX3CR1^+^ CD8^+^ T cells to the intestinal mucosa, establishing a sustained inflammatory niche. Comparative analyses by Lopez et al. further underscore distinct immune profiles between ICI-colitis and classical inflammatory bowel diseases. They reported that although CD8^+^ T-cell infiltration patterns are broadly similar between ICI-colitis and ulcerative colitis (UC), the abundance and activation levels of TRMs are significantly greater in ICI-colitis (46). Conversely, UC lesions predominantly harbor B cells and plasmablasts, highlighting fundamental differences in the immune mechanisms that drive these two forms of intestinal inflammation (30). Mucosal-associated invariant T (MAIT) cells represent another critical cytotoxic T-cell subset involved in ICI-induced colitis. These cells are activated primarily by microbial-derived riboflavin metabolites, which express high levels of inflammatory and cytotoxic markers such as CCL4, GZMB, and the intestinal homing receptor S1PR5. Increased activation of MAIT cells strongly correlates with more severe clinical outcomes, underscoring their role in perpetuating intestinal inflammation (31). Within the CD4^+^ T-cell population, Th17-like cells, characterized by elevated IL-17A and CXCL13 expression, significantly expand during active colitis, likely driving further immune recruitment and inflammation. Interestingly, despite an overall increase, Tregs demonstrate functional impairment and an altered phenotype, as evidenced by upregulated OX40/TNFRSF4 and GITR/TNFRSF18 expression. OX40 and GITR are key Treg-associated costimulatory receptors. GITR is constitutively expressed at high levels on Tregs, whereas OX40 expression is largely TCR signal-dependent and is transiently induced within 24–72 h after TCR engagement, although it can be detected across Treg subsets. The functions of OX40 and GITR are highly context-dependent, with their net effects shaped by local immune activation, ligand availability, and cytokine profiles. Under physiological or controlled inflammatory conditions, these TNFRSF receptors support Treg survival and function by maintaining FoxP3 stability, limiting apoptosis, and promoting local expansion, thereby reinforcing Treg-mediated immunosuppression. However, in the highly inflammatory milieu induced by ICIs, OX40 and GITR signaling may instead drive Tregs from a stable suppressive state toward a dysfunctional or even pro-inflammatory phenotype through TRAF-dependent pathways, including NF-κB and MAPK signaling (47). Single-cell sequencing provides direct support for this transition. In colonic tissues from patients with ICI-associated colitis, a TNFRSF18-high Treg subset showed marked transcriptional reprogramming, whereas a TNFRSF4-high Treg subset displayed clear phenotypic remodeling, including upregulation of Th1-associated genes such as TBX21, IL12RB1, IL12RB2, and CXCR3, as well as other TNF receptor family members (29). These findings suggest that Tregs undergo pathological transcriptional remodeling in ICI-associated colitis, whereby impaired suppressive function may paradoxically contribute to, rather than restrain, intestinal inflammation (29, 31, 48).

Myeloid cell polarization further shapes the inflammatory milieu in ICI-induced colitis. ScRNA-seq identified a distinct proinflammatory macrophage subpopulation(CD68^high^ CXCL10^high^ macrophages). These cells not only secrete key cytokines such as IL-1β and TNF-α but also activate the NLRP3-PYCARD inflammasome through JNK signaling, thereby amplifying innate immune responses. Moreover, they exhibit high expression of interferon-stimulated genes and chemokines such as CXCL9 and CXCL10, recruiting CXCR3^+^ effector T cells to mucosal lesions, thereby amplifying a feed-forward inflammatory cascade and ultimately exacerbating epithelial damage (29, 44).

Moreover, intestinal dysbiosis has emerged as an important environmental driver influencing local immune dynamics (49). Microbial composition characterized by elevated abundance of Alistipes and reduced abundance of protective Lachnospiraceae promotes IFN-γ-producing effector T-cell expansion through Toll-like receptor signaling. Concurrently, the depletion of beneficial commensals such as Roseburia reduces short-chain fatty acid (SCFA) production, impairing mucosal barrier function and intensifying inflammation.

TCR-seq analysis further elucidates the clonal dynamics associated with treatment response and disease persistence. High overlap of Clonotype-1 and Clonotype-3 CD8^+^ T-cell clones between the peripheral blood and inflamed colon suggests transcirculatory migration of ITGB2^high^ CD8^+^ T cells (29). Selective depletion of Clonotype-1 following anti-TNF-α (infliximab) treatment, alongside persistence of Clonotype-40, underscores the potential involvement of Clonotype-40 in recurrence risk (31).

Taken together, the cytotoxic effects of Teff and TRM cells, MAIT cell activation, Th17/Treg imbalance, and inflammatory loops driven by proinflammatory myeloid cells, exacerbated by microbial dysbiosis, collectively compromise the intestinal epithelial barrier and perpetuate the pathogenesis of ICI-induced colitis.

#### 2.3.2 Potential therapeutic targets

The pathogenesis of ICI-induced colitis involves multidimensional immune dysregulation, and intervention strategies targeting different segments have synergistic therapeutic potential (**Table 1**).

**Table 1:** Therapeutic targets for ICI-related colitis.

Target category	Specific targets	Intervention strategies
T-cell regulation	•JAK-STAT pathway •CTLA-4 •CXCL13-CXCR5 axis	• Tofacitinib (JAK inhibitor) • Abatacept (CTLA-4 agonist) • CXCL13/CXCR5 antagonists
Microbial-immune axis	• Gut microbiota • MR1-MAIT axis	• FMT • Anti-MR1 antibody
Inflammatory mediators	• IL-1β • JNK pathway	•Canakinumab (IL-1β antagonist) •JNK inhibitors

In terms of T-cell regulation, a JAK-STAT inhibitor (tofacitinib) can effectively reduce the expression of TRM activation markers (CD38^+^ HLA-DR^+)^ by inhibiting STAT1 phosphorylation in CD8^+^ TRM cells and interfering with IFN-γ production, and its fast-relieving effect on hormone-refractory colitis has been confirmed in clinical studies (30, 50). Moreover, CTLA-4 agonists (abatacept) regulate immune homeostasis by restoring Treg function, but attention should be given to their potential ability to suppress tumor immunity (51). Combination therapy targeting T cell migration and homing exhibits synergistic effects. The combination of vedolizumab and S1PR modulators (ozamodes) can doubly inhibit the intestinal homing and residence of T cells (44).Targeting the CXCL13-CXCR5 axis could potentially contribute to limiting immune cell recruitment and tertiary lymphoid structure (TLS) formation (29). As CXCL13 is a central chemokine involved in TLS formation during irAE pathogenesis (52). By binding to CXCR5, which is mainly expressed on B cells, follicular helper T cells, and subsets of dendritic cells, CXCL13 recruits and spatially organizes immune cells, promoting the formation of lymphoid aggregates with germinal center-like features (53, 54). In inflammatory bowel disease models, strategies targeting CXCL13 have shown certain therapeutic potential. Research has confirmed that CXCL13 gene knockout mice exhibit reduced inflammatory response, decreased levels of pro-inflammatory cytokines, and better survival rates in DSS induced colitis (55). Although there is currently no research on the therapeutic effect of directly blocking the CXCL13-CXCR5 axis in animal models of colitis induced by immune checkpoint inhibitors, we speculate that CXCL13 may play a particularly important pathological role in ICI-associated colitis. CXCL13 gradients at inflamed sites may direct CXCR5^+^ B cells and T cells toward the colonic mucosa and facilitate the development of mature TLS. These structures may sustain intestinal inflammation by supporting local antibody production, complement activation, and T-cell reactivation, thereby converting an initially T-cell-driven response into a more persistent autoimmune-like process. Clinically, this sustained immune activation may contribute to severe manifestations, including diarrhea, abdominal pain, and, in rare cases, intestinal perforation. Preclinical studies suggest that blockade of this pathway with anti-CXCL13 monoclonal antibodies, such as MAb 5261, or CXCR5 antagonists, such as PF-06835375, can reduce immune-cell recruitment, disrupt TLS organization, and inhibit *de novo* TLS formation (56, 57).

The regulation of the microbial-immune axis provides new directions for personalized therapy. Fecal microbiota transplantation (FMT) restores protective flora (Roseburia), repairs intestinal barrier function, and inhibits MAIT overactivation, whereas anti-MR1(MHC class I-related protein 1) antibodies block colony metabolite-driven activation of MAIT cells (58), and specific blockade of MR1-MAIT cell interactions (anti-MR1 antibody) attenuates colony-driven inflammatory responses (9, 59).

At the level of inflammatory mediators, the IL-1β antagonist canakinumab reduces intestinal barrier damage by targeting NLRP3 inflammatory vesicles (45, 60). Moreover, inhibitors of the JNK pathway inhibit the production of IL-1β and the chemokine CXCL9/10 by myeloid cells, resulting in a multilayered anti-inflammatory network (29).

#### 2.3.3 Biomarkers for diagnosis and monitoring

The identification and dynamic monitoring of biomarkers across peripheral blood, intestinal tissue, and the gut microbiota offer valuable tools for the precise diagnosis and management of ICI-induced colitis (**Table 2**).

**Table 2:** Biomarkers for ICI-related colitis.

Source	Specific biomarkers
Peripheral blood	• GZMK^+^ effector CD8^+^ T cells • Clonotypedynamics (Clonotype-3/Clonotype-40)
Serum	• Improve CXCL9/CXCL10 • IL-17A/GZMB
Gut microbiome	• Alistipes-to-Roseburia ratio • SCFA levels

In the peripheral compartment, the abundance of GZMK^+^ effector CD8^+^ T cells (Teff^GZMK^) and the persistence of specific TCR clonotypes, such as Clonotype-3 contraction and Clonotype-40 persistence, have been proposed as noninvasive indicators of disease activity and recurrence risk (31).

In the serum, elevated levels of CXCL9 and CXCL10 reflect the degree of IFN-γ pathway activation and are strongly correlated with endoscopic inflammation scores (44). Additionally, increased expression of IL-17A and GZMB may indicate increased activity of Th17-like and MAIT cells, serving as predictors of treatment resistance (30).

Gut microbiota-derived biomarkers have also emerged as critical indicators. The Alistipes-to-Roseburia ratio is correlated with MAIT cell activation and mucosal inflammation. Moreover, tracking short-chain fatty acid (SCFA) levels can inform the efficacy of microbiota-targeted interventions, such as FMT or dietary modulation (44). Collectively, the integration of these multidimensional biomarkers—spanning cellular, molecular, and microbial levels—can facilitate the transition from empirical therapy to precision-guided management of ICI-induced colitis (45).

## 3 ICI-associated cardiotoxicity

### 3.1 Clinical manifestations and diagnosis

Cardiotoxicity is a rare but potentially fatal irAE of ICIs. Symptoms are often nonspecific and may appear weeks to months after treatment initiation, leading to diagnostic delays. Most commonly presenting as myocarditis (61, 62), but also includes pericarditis, arrhythmias, acute coronary syndrome, and vasculitis (62, 63). The incidence remains uncertain due to underreporting and a lack of standardized diagnostic criteria, although the number of cases has increased with increasing ICI use (64). PD-1/CTLA-4 combination therapy is among the strongest risk factors for ICI-associated myocarditis, substantially increasing its incidence, clinical severity, and fatality rate (65). The PD-1/PD-L1 pathway contributes to immune homeostasis in the heart by restraining local T-cell activation. Its blockade may therefore release myocardial immune tolerance and promote the activation of cardiac-reactive T cells. In the setting of combination therapy, CTLA-4 blockade may further expand autoreactive T-cell clones in lymphoid tissues, whereas PD-1 blockade removes inhibitory signals within the myocardium, together generating a synergistic autoimmune attack (66). Consistently, animal studies have shown that combined PD-1 and CTLA-4 blockade can induce fatal myocarditis characterized by marked CD8^+^ T-cell and macrophage infiltration, supporting this proposed mechanism (67). Early recognition is crucial for improving outcomes (68). Diagnosis is based on clinical presentation, cardiac biomarkers, ECG, echocardiography, and cardiac magnetic resonance imaging (CMR), the current gold standard for tissue characterization. However, CMR may be inconclusive in early disease. 68Ga-DOTATOC PET/CT has shown promise in early detection but requires further validation (69). Disease management depends on disease severity. ICIs should be discontinued immediately upon diagnosis. Mild cases may be monitored with supportive care, whereas moderate to severe myocarditis typically requires high-dose corticosteroids. In refractory cases, agents such as mycophenolate mofetil, IVIG, or infliximab may be considered. Pretreatment cardiac risk assessment and close monitoring in high-risk patients are recommended (70).

### 3.2 Pathophysiological mechanisms

The pathogenesis of ICI-MC has not been fully elucidated but is widely attributed to dysregulated immune responses following immune checkpoint blockade (71, 72). By increasing inhibitory signals on T cells, ICIs promote unchecked activation and expansion of CD4^+^ and CD8^+^ T cells, which infiltrate cardiac tissue and initiate cytotoxic attacks on cardiomyocytes, leading to myocarditis and related complications. In parallel, ICIs may disrupt immune tolerance, triggering the production of cardiac autoantibodies such as anti-acetylcholine receptor and anti-rhabdomyosin antibodies. These autoantibodies can activate the complement system or mediate antibody-dependent cytotoxicity, further contributing to myocardial injury and electrical instability, such as arrhythmias. Moreover, activated immune cells release high levels of proinflammatory cytokines-most notably IFN-γ, IL-1β, and TNF-α-which exacerbate tissue inflammation, promote cardiomyocyte apoptosis, disrupt the myocardial structure, and impair electrophysiological function (73). In addition, unlike ICI-colitis, the role of Tregs in myocarditis is still unclear. The number of FoxP3^+^ Tregs is reduced (74). Although studies have mentioned impaired Tregs function in animal models of myocarditis (75), functional inhibitory analysis data directly conducted in ICI myocarditis patients is still limited.

### 3.3 Insights from single-cell RNA sequencing

#### 3.3.1 Immune microenvironment heterogeneity and signaling cascades

ScRNA-seq has enabled high-resolution profiling of immune cell populations in ICI-MC patients, revealing complex and dynamic interactions among T cells, myeloid cells, and stromal components (76).

At the T-cell level, cardiac-infiltrating CD8^+^ T cells display pronounced clonal expansion and functional heterogeneity. Effector and proliferative subsets express high levels of cytotoxic mediators (GZMB and PRF1) and tissue-resident markers (CXCR6 and ITGA1), which directly mediate cardiomyocyte injury. Strikingly, ~63% of these cells exhibit TCR clonotypes specific for the cardiac antigen α-myosin, and adoptive transfer of such clones has been shown to induce fatal myocarditis in murine models (77–79). Despite expressing exhaustion markers (PD-1, LAG3, HAVCR2), these CD8^+^ T cells retain effector functions and continuously secrete IFN-γ and chemokines (CCL4, CCL5), thereby sustaining immune cell recruitment and perpetuating local inflammation (80, 81). Furthermore, a marked deficiency of γδT cells and MAIT cells in the circulation of affected patients may compromise the immunoregulatory balance and exacerbate pathology (79).

Myeloid cell polarization further amplifies the inflammatory cascade. A distinct subset of NLRP3^+^ IL-1β^+^ inflammatory macrophages whose abundance correlates with serum troponin levels and adverse clinical outcomes (82), has been identified via scRNA-seq; these macrophages promote CD8^+^ T-cell activation and neutrophil recruitment via the secretion of IL-1β, TNF-α, and CXCL9/10 (78, 83, 84). Another subset, characterized by high expression of complement components (C1qA/B/C) and Fc receptors (FCγRIIIa), contributes to myocardial injury through classical complement activation and membrane attack complex (MAC) formation. Trajectory inference suggests that CCR2^+^ monocytes differentiate into terminal CXCL9^+^CXCL10^+^ macrophages under IFN-γ stimulation, a process that can be attenuated by CCR2 blockade (83). These findings underscore the central role of the monocyte–macrophage axis in shaping the inflammatory milieu.

Spatial transcriptomics integrated with scRNA-seq further elucidates intercellular regulatory circuits within the inflamed myocardium. CD8^+^ T cells and macrophages form a self-reinforcing loop via the IFN-γ–CXCL9/10–CXCR3 axis, driving chronic inflammation (81, 83, 85). Under inflammatory stimulation, fibroblasts, acquire a CXCL9^+^HLA-DRA^+^ phenotype and contribute to immune cell recruitment and antigen presentation through interactions with dendritic cells (86). In addition, venous endothelial cells specifically express ACKR1, which interacts with myeloid cells and CXCL9+ fibroblasts through the CXCL signaling pathway, promoting the recruitment of immune cells (87).

Collectively, these insights delineate the cellular and molecular heterogeneity of the ICI-MC immune microenvironment, offering a mechanistic rationale for targeted therapeutic interventions.

#### 3.3.2 Potential therapeutic targets

Therapeutic strategies for ICI-MC can be categorized into three major domains: modulation of pathogenic immune cells, interruption of inflammatory signaling pathways, and remodeling of the cardiac immune microenvironment (**Table 3**).

**Table 3:** Therapeutic targets for ICI-related cardiotoxicity.

Target category	Specific targets	Intervention strategies
Cytotoxic T-cell activity	•Reduce GZMB/PRF1 • CCR5/CXCR3	• Anti-CD8 antibodies /CAR-T cell • Maraviroc (CCR5 antagonist)
Myeloid-driven inflammation	• IL-1β/TNF-α • JAK-STAT pathway • Complement cascade	• Infliximab (anti-TNF-α) • Ruxolitinib (JAK inhibitor) • Eculizumab (anti-C5)/anti-CD16a
Cardiac microenvironment	• Integrin signaling • Gut-heart axis	• Natalizumab (integrin inhibitor) • Probiotics/FMT

Selectively targeting cytotoxic T cells or their effector functions is a key strategy in ICI-MC therapy. The selective depletion of overexpanded pathogenic T-cell clones may be achieved via the use of anti-CD8 antibodies or engineered CAR-T cells that target specific TCR clonotypes. To inhibit cardiac infiltration, blocking chemokine-guided trafficking with CCR5 antagonists (e.g., maraviroc) or CXCR3 inhibitors has shown promise in preclinical models by reducing effector T-cell recruitment and local inflammation (77, 79, 81). Moreover, by neutralizing GZMB or PRF1 with monoclonal antibodies to directly block the tissue damage mediated by this pathway (88), rather than depleting or completely inhibiting CD8+ T cells, it is expected to reduce the damage to target organs such as the heart while maximally preserving the T-cell-mediated anti-tumor immune response. It should be emphasized that this strategy requires more precise intervention: its ultimate goal is not broad-spectrum inhibition, but rather to gain a time window and provide synergistic support for the subsequent specific depletion of pathogenic CD8+ T cells based on TCR clonality, single-cell transcriptome characteristics, or tissue-resident markers.

The regulation of myeloid-driven inflammation represents another therapeutic avenue. IL-1β receptor antagonists and anti-TNF-α monoclonal antibodies (89)(e.g., infliximab) directly suppress pivotal proinflammatory cytokines involved in tissue damage. In addition, inhibition of the JAK-STAT pathway—particularly with ruxolitinib-attenuates IFN-γ–mediated macrophage activation and dampens downstream inflammatory cascades (78, 83). Controlling complement activation can prevent immune-mediated myocardial injury (90). Eculizumab, an anti-C5 monoclonal antibody, blocks the terminal complement cascade and MAC formation (91), whereas therapeutic inhibition of CD16a (FcγRIIIa) can disrupt pathogenic IgG–Fc receptor interactions, limiting antibody-dependent cardiac damage (79).

Remodeling the cardiac microenvironment is also a promising strategy. Inhibition of integrin signaling with natalizumab suppresses the proinflammatory phenotype of cardiac fibroblasts and reduces CXCL9 production, thereby interrupting the IFN-γ–CXCL9/10–CXCR3 amplification loop (87, 92). Furthermore, modulation of the gut–heart immune axis through FMT or probiotic administration has been proposed to restore immune homeostasis and reduce systemic inflammatory tone (93).

#### 3.3.3 Biomarkers for diagnosis and monitoring

Biomarker studies of ICI-MCs have focused on the multidimensional integrated analyses of peripheral circulation, histopathology, and genetic susceptibility (**Table 4**).

**Table 4:** Biomarkers for ICI-related cardiotoxicity.

Source	Specific biomarkers
Peripheral blood	• α-myosin-specific CD8^+^ T cell clones • HLA-A*03:01 allele
Plasma	• S100A8/A9 • Phosphatidylinositol (PI 18:0/18:3)
Cardiac tissue	• IL-18^+^ TNF^+^ myeloid cells • C4d deposition

In the peripheral immune compartment, TCR-seq has revealed α-myosin–specific CD8^+^ T-cell clones in circulation, which mirror those infiltrating the myocardium, suggesting a peripheral signature of cardiac-targeted immunity. Moreover, HLA-A*03:01 has emerged as a genetic risk allele, likely enhancing antigen presentation of α-myosin peptides, thereby predisposing individuals to autoreactive T-cell expansion and ICI-MC development (76). Systemically, inflammatory and metabolic biomarkers offer additional insight into disease activity. Elevated serum levels of S100A8/A9, a damage-associated molecular pattern (DAMP), are positively correlated with ICI-MC severity. When assessed alongside dysregulated lipidomic profiles—notably, changes in phosphatidylinositol (PI 18:0/18:3) and specific triglycerides—these markers reflect the interplay between metabolic stress and immune activation, forming a dynamic indicator of pathophysiological shifts (94).

At the tissue level, cardiac biopsy findings further enrich diagnostic and prognostic evaluation. A high abundance of IL-18^+^ TNF^+^ myeloid cells is associated with adverse clinical outcomes, highlighting their contribution to cytokine-driven tissue injury. Additionally, C4d deposition, as detected by immunohistochemistry, provides direct evidence of complement activation, implicating the classical pathway in myocardial damage (78, 81).

Altogether, this multifaceted biomarker framework—encompassing peripheral immune monitoring, tissue-specific pathology, and host genetic factors—enables early detection, facilitates mechanistic understanding, and supports personalized therapeutic decision-making in patients at risk for or presenting with ICI-MC.

##### 3.3.3.1 Comparison of the mechanisms of colitis and cardiotoxicity

**Figure 2:**
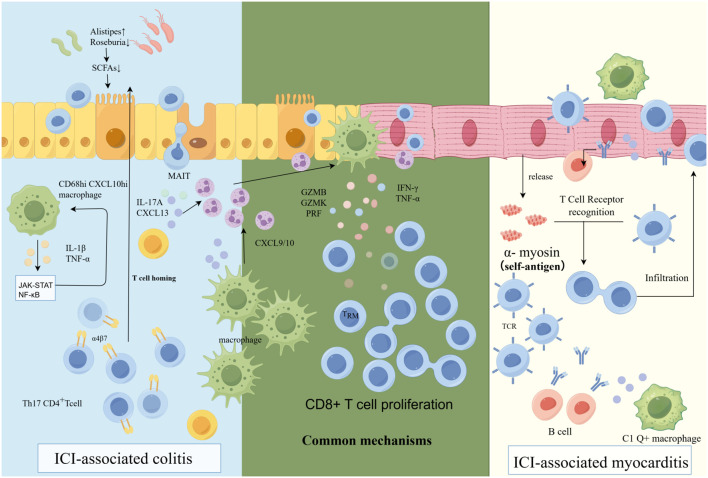
Distinct and shared mechanisms underlying ICI-associated colitis and myocarditis. In colitis, gut microbiota dysbiosis (Alistipes↑, Roseburia↓) leads to reduced short-chain fatty acids (SCFAs), triggering mucosal-associated invariant T (MAIT) cell activation and macrophage recruitment. CD68hi CXCL10hi macrophages secrete IL-1β and TNF-α, activating JAK-STAT and NF-κB signaling. Th17 CD4^+^ T cells, expressing α4β7 integrin, home to the gut mucosa, secreting IL-17A and CXCL13 to promote T cell infiltration and TLS formation. In the shared central panel, macrophages produce CXCL9/10, driving CD8^+^ T cell proliferation and TRM cell activation, with release of cytotoxic effectors (GZMB, GZMK, PRF) and pro-inflammatory cytokines (IFN-γ, TNF-α). In myocarditis, cardiomyocyte injury releases α-myosin (self-antigen), which is recognized by TCR on CD8^+^ T cells, leading to clonal expansion, infiltration, and antibody production by B cells. C1q^+^ macrophages and complement components further amplify cardiac inflammation. This figure emphasizes the convergent role of cytotoxic T cell responses and macrophage-mediated inflammation across distinct irAEs.

ICI-associated colitis and myocarditis share core immune mechanisms centered on clonally expanded CD8^+^ T cells that infiltrate target tissues—the intestinal epithelium or cardiomyocytes—and exert cytotoxic effects via GZMB, PRF1, IFN-γ, and TNF-α. This cytotoxicity is amplified by activated myeloid cells (macrophages and monocytes) through IL-1β and TNF-α release, triggering NF-κB and JAK-STAT signaling and sustaining local inflammation. Both conditions involve chemokine-mediated recruitment (e.g., CXCL9/10–CXCR3) and persistent activation of tissue-resident immune cells (colonic TRMs, cardiac CXCL9^+^ macrophages), contributing to disease chronicity. However, distinct organ-specific pathways shape their pathogenesis (**Figure 2**). Colitis is modulated by gut-homing mechanisms (α4β7 integrin), Th17 polarization (IL-23/STAT3 axis), and microbiota–MAIT cell interactions. In contrast, myocarditis involves T-cell responses to cardiac autoantigens (e.g., α-myosin), complement activation (C4d), and humoral factors (e.g., anti-rhabdomyosin antibodies). The synergy between IFN-γ and IL-1β is especially prominent in the cardiac microenvironment. Therapeutic strategies differ accordingly: colitis responds to microbiota modulation and antihoming therapies (e.g., vedolizumab), whereas myocarditis requires blockade of autoimmune and complement pathways (e.g., IL-1β or C5 inhibitors). These insights emphasize the value of single-cell profiling in delineating shared and tissue-specific immune circuits, and highlight opportunities for developing cross-organ or tailored interventions (**Figure 2**).

## 4 Application of scRNA-seq in ICI-associated pneumonitis and other irAEs

In ICI-associated pneumonitis (ICI-P), scRNA-seq has clarified the previously debated roles of the Th1 and Th17 pathways by identifying a distinct subset of pathogenic Th17.1 cells. These cells coexpress Th1 (TBX21) and Th17 (RORγt) transcriptional markers, secrete both IFN-γ and IL-17A, and exhibit increased cytotoxicity, contributing to pulmonary inflammation. Notably, Th17.1 cells engage in a proinflammatory feedback loop with monocytes via the GM-CSF/IL-23 axis: GM-CSF drives monocyte polarization toward an M1-like phenotype, while IL-23 secreted by monocytes further activates Th17.1 cells and enhances their pathogenicity (95). In severe ICI-P, a distinct aberrant basaloid epithelial cell population, marked by SOX9(SRY-box transcription factor 9) overexpression, has been identified. These cells secrete CXCL3 and CXCL5, promote the recruitment of CXCR2^+^ neutrophils, and act in concert with CD8^+^ effector T cells to create a proinflammatory lung microenvironment (96). In addition, Serum ELISA revealed elevated IgG autoantibodies against surfactant protein B (SP-B), which may serve as disease biomarkers, in patients with ICI-P. These SP-B-specific IgG autoantibodies co-occur with, and correlate positively with, circulating SP-B–specific CD4^+^ IFN-γ^+^ T cells. The expanded SP-B-specific CD4^+^ T-cell clones exhibit an activated, proinflammatory transcriptional profile—including high TNF expression-and clonally expand in peripheral blood preceding ICI-P onset, implicating these clones as direct mediators of lung-localized immunopathology (97). Together, these findings suggest that therapeutic strategies targeting the GM-CSF/IL-23 axis or the SOX9–CXCL3/5–CXCR2 pathway may offer novel avenues for treating ICI-P.

These SP-B-specific IgG autoantibodies co-occur with, and correlate positively with, circulating SP-B–specific CD4^+^IFN-γ^+^ T cells. The expanded SP-B-specific CD4^+^ T-cell clones exhibit an activated, proinflammatory transcriptional profile-including high TNF expression-and clonally expand in peripheral blood preceding ICI-P onset, implicating these clones as direct mediators of lung-localized immunopathology.

In ICI-associated hepatitis, CD8^+^ effector memory T cells in the peripheral blood are the primary mediators of IFN-γ pathway activation, whereas CD16^+^ monocytes contribute to systemic immune-metabolic dysregulation, including perturbed alanine metabolism (98). TCR-seq further revealed the clonal expansion of antigen-specific T cells, implicating a targeted immune response in hepatic injury.

In ICI-induced erythema nodosum-like skin toxicity, scRNA-seq revealed an expansion of GZMB^+^ cytotoxic CD8^+^ T cells with shared TCR clonotypes detected in both the skin and peripheral blood, suggesting systemic immune involvement (99). These findings were accompanied by increased IFN signaling in monocyte subsets and downregulation of immunosuppressive genes such as S100A8/A9, which may exacerbate local inflammation.

Collectively, these studies underscore the utility of scRNA-seq in mapping immune cell heterogeneity, identifying organ-specific immune effectors, and uncovering potential biomarkers and therapeutic targets across diverse irAE phenotypes (**Table 5**).

**Table 5:** Therapeutic targets and biomarkers for other irAEs.

Toxicity type	Therapeutic targets		Biomarkers	
	Target	Specific targets	Source	Specific biomarkers
Pneumonitis	Pathogenic Th17.1 cells	• GM-CSF/IL-23 axis • SOX9-CXCL3/5-CXCR2 pathway	Serum	Anti-SP-B IgG autoantibodies
Hepatitis	• CD8^+^ T-cell activation • Monocyte metabolic reprogramming	• IFN-γ signaling • CD16^+^ monocyte activity	Peripheral blood	• IFN-γ pathway activation ( STAT1 phosphorylation) • Altered alanine metabolism
Erythema nodosum-like skin toxicity	Cytotoxic T-cell signaling immunosuppressive gene regulation	• IFN signaling • S100A8/A9 downregulation	• Skin •peripheral blood	• GZMB^+^ cytotoxic CD8^+^ T-cell expansion • IFN signature upregulation (MX1, ISG15)

## 5 Summary and outlook

**Figure 3:**
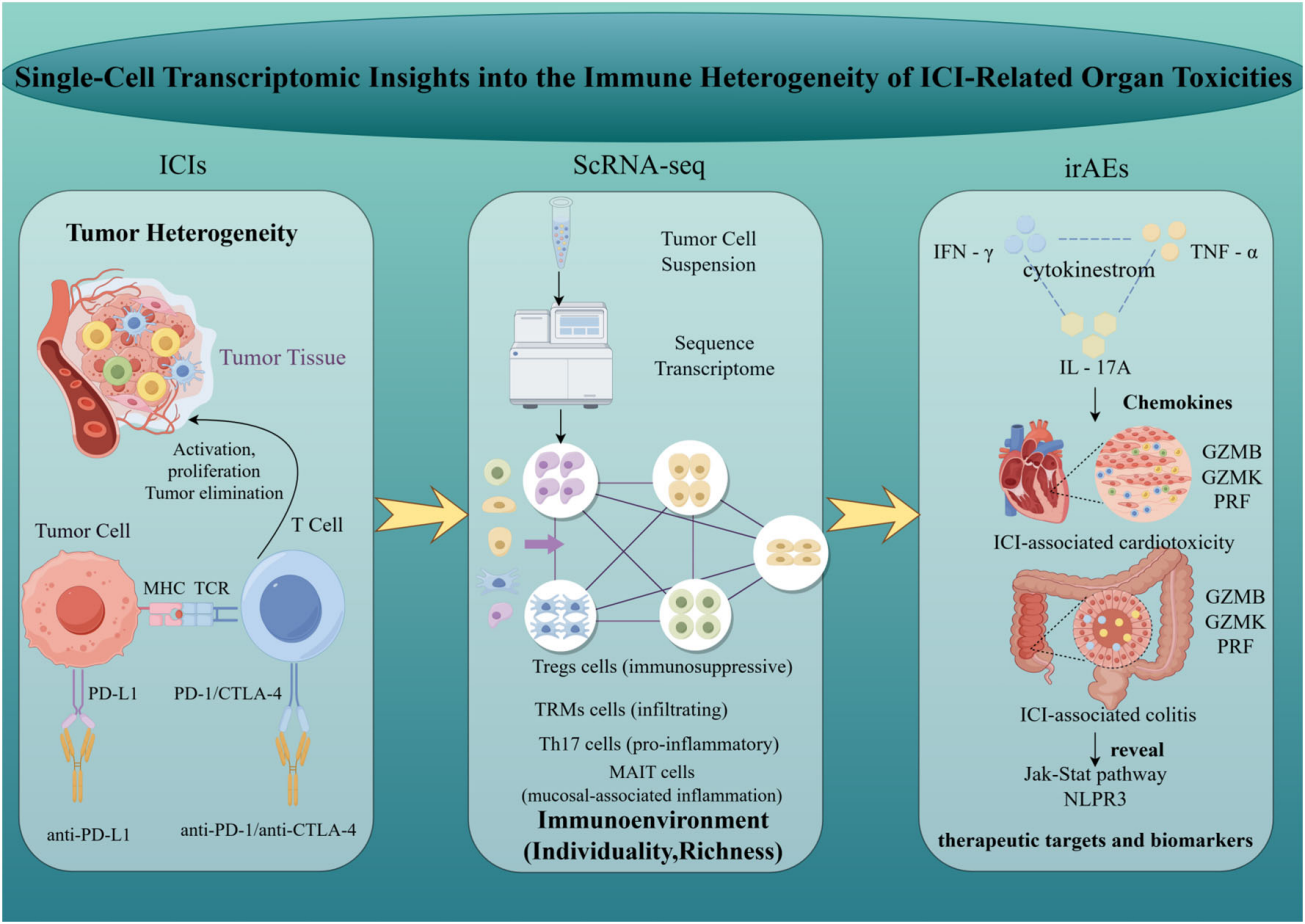
ScRNA-seq reveals the immune heterogeneity of ICI-related organ toxicities. ICIs activate T-cell anti-tumorimmunity by blocking the PD-1/PD-L1 or CTLA-4 pathway; scRNA-seq technology is employed to analyze the cellular heterogeneity of the immune microenvironment from tumor suspensions, distinct pro-inflammatory and cytotoxic T cell subsets infiltrate affected organs, and shape the local immune environment. These cell-type-specific profiles highlight the Jak-Stat pathway, NLRP3 inflammasome, as potential biomarkers and therapeutic targets for individualized management of ICI-related cardiotoxicity and colitis.

ICI therapy enhances T cell-mediated antitumor immunity by releasing inhibitory immune checkpoints. However, the heterogeneity of the tumor and host immune microenvironment also predisposes some patients to severe organ-specific irAEs. In affected tissues, distinct pro-inflammatory and cytotoxic T-cell subsets infiltrate target organs and reshape the local immune landscape (**Figure 3**). ScRNA-seq has advanced our understanding of ICI-associated toxicity by revealing cellular heterogeneity, intercellular networks, and regulatory dynamics, thereby supporting the discovery of early biomarkers and therapeutic targets.

Despite these advances, the translation of scRNA-seq findings in ICI-associated toxicity remains constrained by challenges in sample acquisition, data analysis, and clinical application. First, clinical samples are often scarce and difficult to obtain, particularly for organs requiring invasive biopsy, such as the heart. Interpatient heterogeneity in tumor type, ICI regimen, disease course, and treatment history further complicates multicenter and retrospective analyses. In addition, the requirement for high-quality fresh tissues limits the feasibility of large-scale studies. Second, scRNA-seq data analysis requires substantial bioinformatics expertise. Technical noise, sparsity, and batch effects hinder cross-platform integration, while the lack of spatial information limits the interpretation of local pathological structures, such as focal myocarditis or colonic cryptitis (100). Third, clinical translation remains limited by high costs, infrastructure requirements, difficulties in biomarker validation, and insufficient causal inference, as static single-cell profiles alone cannot distinguish pathogenic drivers from downstream consequences.

To address these limitations, future research should aim to integrate spatial transcriptomics and multi-omics technologies, develop more advanced data integration algorithms, and combine longitudinal clinical cohorts and functional validation experiments to achieve a comprehensive analysis of the mechanisms of ICI organ toxicity. This will provide a solid scientific basis for the development of precise diagnostic markers and intervention targets. In-depth exploration in this field will not only help improve the quality of life and prognosis of cancer patients but also offer valuable paradigms for understanding the pathogenesis of autoimmune diseases in humans.
